# Supplementary material for: The genome sequence of the Welsh wave moth, Venusia cambrica Curtis, 1839
Source: Wellcome Open Res. 2024 Dec 2;9:703. [Version 1] doi: 10.12688/wellcomeopenres.23395.1 (PMC11822252; doi:10.12688/wellcomeopenres.23395.1)
Supplement: Peer review history

# Reviewer report: Reviewer response for version 1

**DOI:** 10.21956/wellcomeopenres.25800.r116699

**Published:** 2025-02-12

**Authors:** Simon T Segar

**Affiliations:** 1 Harper Adams University, Newport, UK

Then is manuscript provides a report of a high quality genome of *Venusia cambrica*. Along with other species sequenced by the Darwin Tree of Life Consortium this represents a highly valuable resource. The field and lab work are rigorous and the data are shared appropriately. There is a lot of detail around analysis and the work is repeatable. Other reviewers have also given suggestions around this area. 

 I have a few relatively minor suggestions and comments.

 1. In the background section the authors could add some examples on ecological applications, there are lots, but otherwise this sounds rather an after thought.

 2. "Assembly errors were corrected by manual curation, including 10 missing joins or mis-joins and 1 haplotypic duplications", no 's' needed. Spell numbers under ten in full.

 3. Table 2. Include mtDNA and rDNA statistics in table or elsewhere?

 4. In the last section of the text it might be good to have a sentence coming back to ecology?

Are sufficient details of methods and materials provided to allow replication by others?

Yes

Is the rationale for creating the dataset(s) clearly described?

Yes

Are the datasets clearly presented in a useable and accessible format?

Yes

Are the protocols appropriate and is the work technically sound?

Yes

Reviewer Expertise:

Molecular ecology, entomology and community ecology.

I confirm that I have read this submission and believe that I have an appropriate level of expertise to confirm that it is of an acceptable scientific standard.

# Reviewer report: Reviewer response for version 1

**DOI:** 10.21956/wellcomeopenres.25800.r116239

**Published:** 2025-01-21

**Authors:** Yu-Feng Huang

**Affiliations:** 1 National Chung Hsing University, Taichung, Taiwan

This manuscript presents the chromosomal-level genome assembly of *Venusia cambrica* (Welsh Wave), a moth species in the family Geometridae, sequenced as part of the Darwin Tree of Life Project. Using high-fidelity Pacific Biosciences long reads and Hi-C chromosomal conformation data, the authors achieved an assembly spanning 470.40 Mb with 99.95% assigned to 38 chromosomal pseudomolecules, including the Z sex chromosome. The assembly exhibits high completeness (BUSCO score: 98.1%, QV: 62.3) and includes a 16.44 kb mitochondrial genome.

 Annotation identified 17,931 protein-coding genes and 18,070 transcribed mRNAs, revealing insights into the species’ genetic structure and organization. The manuscript outlines robust methodologies for sample collection, DNA/RNA extraction, sequencing, and assembly, integrating tools like Hifiasm, purge_dups, and YaHS, with curation supported by PretextView and BlobToolKit. This high-quality genome resource has broad implications for evolutionary and ecological studies in Lepidoptera and aligns with the broader goals of the Darwin Tree of Life Project.

 However, there are several issues that should be addressed:

1. Mitochondrial Genome Length
  - In describing the length of the mitochondrial genome, it would be more precise to show the full length (rather than a floating-point approximation) in both the abstract and Table 3.
  - Additionally, it is worth noting that the mitochondrial genome is significantly shorter than the autosomes and sex chromosomes.
2. Mitogenome Annotation Tool
  - The manuscript states that genome annotation was performed using BRAKER2; however, it does not clearly specify which tool was used for annotating the mitochondrial genome.
3. Data Analysis Workflow
  - It would be beneficial to include a data analysis workflow alongside the information presented in Table 4. This would provide readers with a more comprehensive overview of the methods used.

Are sufficient details of methods and materials provided to allow replication by others?

Yes

Is the rationale for creating the dataset(s) clearly described?

Yes

Are the datasets clearly presented in a useable and accessible format?

Yes

Are the protocols appropriate and is the work technically sound?

Yes

Reviewer Expertise:

bioinformatics, computational biology, cancer bioinformatics, genome assembly and annotation

I confirm that I have read this submission and believe that I have an appropriate level of expertise to confirm that it is of an acceptable scientific standard, however I have significant reservations, as outlined above.

# Reviewer report: Reviewer response for version 1

**DOI:** 10.21956/wellcomeopenres.25800.r116245

**Published:** 2025-01-06

**Authors:** Oliver Hawlitschek

**Affiliations:** 1 University of Zurich, Zurich, Switzerland

The report sounds mostly complete, clear, and straightforward to me. The figures look good to me. I am not an expert on genome sequencing and assembly, so I focused on the organismic part. I just have two minor comments:

 1. Figure 2: The sentence “The BlobToolKit snail plot […]” is given twice.

 2. Methods: To match the "Legal and Governance" part, I wonder if it makes sense to describe the legal protection status of the target species in the UK and describe if any permits were necessary.

 3. The methods are described in detail separately for PacBio, Hi-C, and RNA, up to the sub-heading “Genome assembly, curation and evaluation”. After that, it is only about Hi-C. Is the rest summed up under “primary contigs”, or under primary and secondary analysis“ in the “Library preparation and sequencing” part? Perhaps it is worthwhile adding a very short description of what happened to the PacBio and RNA reads.

Are sufficient details of methods and materials provided to allow replication by others?

Partly

Is the rationale for creating the dataset(s) clearly described?

Yes

Are the datasets clearly presented in a useable and accessible format?

Yes

Are the protocols appropriate and is the work technically sound?

Yes

Reviewer Expertise:

Phylogenomics and population genomics

I confirm that I have read this submission and believe that I have an appropriate level of expertise to confirm that it is of an acceptable scientific standard, however I have significant reservations, as outlined above.
